# Show Your Pride? First-Generation College Student Experiences with Academic Achievement

**DOI:** 10.1007/s42761-025-00309-w

**Published:** 2025-06-06

**Authors:** Hugo Sanchez Hernandez, Jorge Castro, Belinda Campos

**Affiliations:** 1 Department of Psychology, University of California, Los Angeles, CA USA; 2 Department of Psychological Science, University of California, Irvine, CA USA; 3 Department of Chicano/Latino Studies, University of California, Irvine, CA USA

**Keywords:** Achievement, Capitalization, Pride, Gratitude, First-generation college students

## Abstract

Does upward social mobility shape how individuals navigate emotion-laden experiences? The current study examined this question in the context of academic achievement in a sample of first-generation college students. As the first in their families to attend university, this is a group that finds themselves in university environments that encourage open expression of individual achievement, but who typically come from sociocultural backgrounds where there is wariness of the social distancing that can be created by open expression of individual achievement. Qualitative methodology, which allows for in-depth exploration that can generate novel insights and advance theory, was used to study how first-generation college students at the graduate level (*N* = 32) navigate the emotions that come with academic achievements across academic and family contexts. Thematic analysis of the participant qualitative interview corpus revealed two novel findings. First, participants expressed feelings of both pride and gratitude that included appreciation for those who helped them reach their achievements. Second, participants engaged in *capitalization regulation*—a term that we introduce to describe the thoughtful consideration of how and with whom to share good news with across one’s different social contexts so as to not evoke negative social consequences. These findings contribute to the field’s growing understanding of emotion and social class in an important context, first-generation college students, in which these factors are salient. Implications for understanding the complex emotional experiences associated with upward social mobility are discussed.

**Supplementary Information:**

The online version contains supplementary material available at 10.1007/s42761-025-00309-w.

Upward social mobility, the experience of transitioning from a lower to a higher socioeconomic status, can shape emotion experiences in ways that are important for navigating distinct status worlds. First-generation college students (FGCS) who come from working-class home environments with interdependent norms may experience challenges with navigating the independent social norms of university environments (e.g., Covarrubias & Fryberg, 2015; Vasquez-Salgado et al., 2015). While the challenges of straddling different status worlds have been documented, there is less focus on how those challenges extend to the experience of academic achievement, a key marker of status shifting to upward social mobility. In this study, we report a qualitative exploration of how FGCS navigate the emotion-laden experience of academically achieving across their differing socioeconomic status worlds.

## Independent and Interdependent Self-Construals Across Sociocultural Backgrounds

Self-construal, which represents how individuals perceive themselves in relation to others (Markus & Kitayama, 1991), is important for understanding how emotion experiences vary across individuals of differing sociocultural backgrounds. Most work on self-construals has been focused on ethnic forms of culture, with individualistic cultures (e.g., USA) being more strongly defined in terms of the emphasis placed on individual uniqueness (i.e., independent self-construal) and collectivistic cultures (e.g., Asia and Latin America) being more strongly defined in terms of interpersonal relationships (i.e., interdependent self-construal; Markus & Kitayama, 1991; Singelis, 1994; Triandis, 1989). However, self-construals are also relevant for social class forms of culture. Individuals of lower socioeconomic status (SES), who have less opportunities, resources, and individual control over their circumstances, engage in more interdependent behaviors than those of higher SES, including being more helpful towards others and being better at inferring others’ emotions (Galinsky et al., 2006; Keltner et al., 2003; Kraus et al., 2010; Piff & Moskowitz, 2018; Piff et al., 2010, 2012; Stephens et al., 2007). The differences in self-construal characteristics across SES contexts may have implications for norms around emotion experiences that occur on the trajectory towards upward social mobility.

## First-Generation College Students in the University Environment

One context where self-construal values are relevant and which has implications for emotion norms is the university environment. The challenges faced by first-generation college students (FGCS), the first in their families to attend university, when entering middle-class university environments that carry independent values that differ from the interdependent values of their working-class home environments are well documented (e.g., Burgos-Cienfuegos et al., 2015; Chen et al., 2022; Covarrubias & Fryberg, 2015; Covarrubias et al., 2020; Herrmann & Varnum, 2018; Stephens et al., 2012; Vasquez-Salgado et al., 2015). FGCS feel what has been described as a cultural mismatch, a lack of fit between their university and home values (Vasquez-Salgado et al., 2015). They report family achievement guilt over having more opportunities than family, express concerns over becoming different from family, and may experience less social inclusion and belongingness and more negative emotions due to their disconnect (e.g., Baxter & Britton, 2001; Covarrubias et al., 2020; Destin et al., 2017; Wentworth & Peterson, 2001). Despite this, FGCS may still strive to academically achieve because doing so signals their progress towards upward social and educational mobility. However, academic achievements may evoke emotion and expressions of emotion that may be discouraged within FGCS’ interdependent home environments.

### Emotions Associated with Academic Achievement

The lay theory of emotion is important for understanding emotions associated with academic achievement. The theory concludes that independent emotions arise from within the self and reflect independent self-construals while interdependent emotions are felt in the context of collective shared experiences with others and reflect interdependent self-construals (Uchida et al., 2022). Unlike interdependent and others-focused emotions (e.g., gratitude; McCullough et al., 2001), a self-focused emotion like pride (Tracy & Robins, 2004, 2007) is relevant in the university environment that emphasizes individuality, independence, and self-expression (e.g., Stephens et al., 2012). This is consistent with pride being experienced more in higher SES environments with more independent values, wherein individuals more strongly consider achievements to be of their own doing compared to lower SES individuals (Li & Hu, 2021; Piff & Moskowitz, 2018; Xian & Reynolds, 2017). Pride is relevant for how individuals feel after academically achieving and in the context of the common university expectation to make one’s achievements known.

### Capitalizing on Academic Achievements

Socially sharing one’s good news, termed *capitalizing* (Langston, 1994), can be beneficial to well-being (Gable et al., 2004), but it may also bring potential social consequences. Findings show that individuals who capitalize on achievements and express pride can be perceived negatively by others (Kalokerinos et al., 2014), and individuals from interdependent sociocultural backgrounds do express concerns about the interpersonal consequences of capitalizing (Choi et al., 2019). Considering this potential social backlash, FGCS who come from lower SES, interdependent home environments that place more focus on *others* may find it challenging to openly engage in the university norm to capitalize on individual*-*focused achievements that elicit pride and highlight the *self*. FGCS may find ways to manage how they capitalize on academic achievements, but this possibility needs further exploration.

## Current Study

The current investigation used qualitative methodology to generate a needed in-depth understanding of how FGCS navigate the emotional experiences of academic achievement across their family and university environments. This study provided an opportunity for FGCS to discuss how they capitalize on their achievements when straddling two different SES worlds. The study focused on graduate students because they experience a dramatic shift in their SES worlds and have had time to process and have insight into their experience underdoing educational mobility. By exploring academic achievement, the current study advances literature on emotion, interpersonal emotion regulation, and social class by demonstrating how FGCS regulate their emotional experiences while undergoing upward social mobility.

## Method

### Participants

Graduate students (*N* = 32) from a public California university were eligible if they indicated that no parent or guardian of theirs had obtained a four-year college degree. Participants ranged from 22 to 43 years of age (*M*_age_ = 28.50, *SD*_age_ = 4.90). Most identified as women (20 women, 10 men, one as non-binary, and one preferred not to identify) and as Latino/a/x/e (*n* = 21)^1^ and all but one (Master’s level) of the participants were Ph.D. students. Most were born in the USA (*n* = 26) and most indicated being second-generation (i.e., born in the USA and at least one parent or guardian having been born in another country; *n* = 18). Most considered their social class growing up to be lower/working class (*n* = 20) and most reported their family’s combined annual income to be below $50,000 (*n* = 24).

### Positionality

Consistent with the social constructivist research paradigm to be self-reflexive (Tracy, 2020), we considered how researcher backgrounds may influence data collection and analysis. All three authors of the current study identify as Latino/a/x/e FGCS. The lead author developed the interview guide with the help of the third author, conducted all interviews, and, along with an undergraduate research assistant (the second author), engaged in data analysis. The lead author interpreted findings and wrote all sections of the manuscript, with contributions from the second author (in writing discussion section) and the third author (in interpretation of findings and all stages of writing).

As researchers who were FGCS and who are of Latino/a/x/e heritage, an ethnic group that also made up most of our sample, our personal experiences are relevant to this work. The lead and third authors first engaged in discussions around the experience of straddling differing SES worlds early in the first author’s doctoral program. Guided by findings showing cultural differences in the emotion of pride (Sanchez Hernandez et al., 2023), the first and third authors reflected on the possibility of emotion differences stemming from another important sociocultural factor, SES. Specifically, the first and third authors reflected on their own personal experiences as FGCS undergoing upward social mobility in the university environment, first at the undergraduate level and then at the graduate level. Our proximity to the academic, SES, and cultural backgrounds that shape the current study’s sample offered us insight into the challenges that come with navigating academic environments, especially when communicating achievements to family members. This insight informed our research design. While we recognize that our backgrounds created a relatability towards the participants and may have introduced certain biases into our research, we took the necessary steps to minimize bias as much as possible. This included creating a semi-structured interview template that made no direct mention of sociocultural factors. By acknowledging our positionality, we aim to foster transparency and reflexivity in our research.

### Procedure

All measures and procedures were approved by the university’s Institutional Review Board. All research procedures complied with APA ethical standards in the treatment of participants. Data were collected by the lead author through one-on-one semi-structured interviews conducted virtually, via an online video platform, with graduate students who were recruited through emails and electronic flyers circulated with the assistance of the university’s Graduate Division. The coordinator of a university-wide graduate training program shared recruitment materials through an email listserv with graduate students who had been participants of the program. Additionally, emails which included electronic flyers and a google form were shared with several other graduate programs across the university. Interested participants emailed the lead author, who confirmed their eligibility and scheduled a date and time for a virtual interview. At the beginning of the interview, participants again confirmed their eligibility and consented to participate in the study, including the video-recorded interview. After the interview, participants were asked to complete an online demographics questionnaire. For their participation in the study, participants were compensated with a $10 Target gift card.

#### Interviews

One-on-one interviews consisted of open-ended questions about participants’ experiences attaining academic achievements and subsequently sharing their achievements with family members, academic peers, and people outside of academia or family. Interview questions included asking participants to discuss a specific time they achieved academic success, how they felt about the success, with whom they shared it, and how they felt sharing it.^2^ Participant interviews ranged from 18 to 87 min and averaged 45 min in length. All video-recorded interviews were transcribed verbatim. They were first processed through the web-based transcription software Otter before being cleaned by the second author and further finalized by the lead author.

### Data Analysis

The lead author and second author engaged in two different stages of data analysis.

#### First Analysis Stage

The first stage consisted of the initial creation of the codebook. During this phase, the lead and second authors underwent the different phases of thematic analysis outlined by Braun and Clarke (2006)—familiarization with data, generating initial codes, searching for themes, reviewing themes, and defining and naming themes. They first familiarized themselves with the data by reading the same initial interview transcript and independently making notes of what thematic patterns they saw emerging from the data that were related to emotions and experiences in the context of academically achieving. The lead and second authors followed this same procedure with three more transcripts; they independently reviewed the same transcripts and then met one-on-one to discuss the patterns they saw emerging from each of these additional three transcripts. They created a preliminary codebook in which they organized all of the patterns that emerged from the four interviews’ data in order to create higher-order parent themes and subcodes [that fell under their parent themes]. Once this preliminary codebook was created, the lead and second authors reviewed an additional two transcripts, for a total of six transcripts evaluated. They then met to discuss ways in which to combine, merge, and simplify themes and codes.

For determining saturation, the lead and second authors engaged in a procedure outlined by Guest et al. (2020). This procedure involved calculating a saturation ratio through assessing three elements—Base Size, Run Length, and New Information Threshold. Base Size represented the number of codes that were generated through a review and evaluation of an initial set of interview transcripts. As suggested by Guest et al. (2020), the lead author chose to use the initial six interviews reviewed as the base size from which to generate the number of codes that would be used as the denominator in a saturation ratio. Run Length represented the number of codes that were generated through the review and evaluation of each subsequent set of interview transcripts. In other words, the number of codes generated through each subsequent review of new transcripts was used as the numerator in the saturation ratio. As an example of the initial calculation of the saturation ratio, 55 subcodes were generated from the initial six interview transcripts that were reviewed and then 13 new codes were generated from the next set of two interview transcripts that were reviewed. This meant that for the first assessment of saturation, the saturation ratio was calculated as 13 divided by 55, which yielded a percentage of 23.64%. As described by Guest et al. (2020), the goal of this procedure is to eventually yield a saturation ratio of 5% or lower; this is deemed the New Information Threshold, indicating when only 5%, or lower, new codes are generated. Thus, the lead and second authors continued to review new subsequent sets, of two transcripts each, until the number of new codes generated by each subsequent set was small enough to yield a ratio of 5% or lower. This procedure continued until the lead and second authors finally reached saturation after the 14th participant interview, when only one new code was generated by the 13th and 14th transcripts (1/55 yielded a ratio of 1.8%).

Throughout the recurring review of new transcripts for determining saturation, the lead and second authors continued to meet every two weeks after new pairs of transcripts were reviewed (one transcript weekly). Along the way, disagreements were resolved through discussions that included making decisions on the best ways to combine parent themes and subcodes as appropriate. Although saturation was reached with the 14th interview, the lead and second authors did a more streamlined review of an additional 11 transcripts that had not yet been reviewed, chosen at random, in order to ensure a full and thorough review of the data and to confirm that no new themes or codes would be generated. Through the review of these additional eleven transcripts, only one new code emerged that was related to the study aims, and it was added to the codebook. The updated version of the codebook was refined (i.e., themes and codes were revised, merged, and/or combined), and definitions and guidelines on using the codes were added.

#### Second Analysis Stage

The goal of the second stage of data analysis was to determine interrater reliability and code the entirety of the data (i.e., 32 interview transcripts). Within every interview transcript, each response to each specific question was identified as the unit of analysis to be coded, labeled for our study purposes as a “segment.” A segment was longer than one single response in the cases where probing questions were asked or the thematic content of one response extended to the response of a subsequent question. Please see the interview guide for each specific question.

Using the finalized version of the codebook developed in the first stage, which had a total of 33 subcodes that fell under seven parent themes, the lead and second authors independently coded one interview transcript weekly, chosen at random, in order to determine reliability. Coding disagreements and ways to resolve them, including refining coding patterns for future transcripts, were mutually discussed on a weekly basis. In order to ensure reliable assessment of agreement between coders for a representative sample of our data, interrater reliability was calculated after 14 transcripts (44% of data) were coded (95% agreement rate; Cohen’s Kappa of 0.74). Following this, the lead and second authors coded the rest of the data separately following the same timeline of one transcript weekly. Coding for this second stage of data analysis was conducted through the computer qualitative software NVivo (Release 1.7.1; QSR International, 2022), which was also used to calculate the frequencies of subcodes across all data once they were all coded.

To best represent the findings that emerged from the data, two central larger-scale themes are presented in the “Findings” section. These two themes are amalgamations of the subcodes that were identified the most frequently in our participants and that were deemed as best encapsulating the study’s main interest of providing novel insight into FGCS’ emotional experiences in the context of their academic achievement.

### Research Transparency

Due to the qualitative nature of the paper and its potentially identifiable data, data (i.e., transcribed interviews) will not be made available in a repository. Research material (i.e., interview guide) and participant ethnic/racial backgrounds are provided as supplemental material.

## Findings

Two central larger-scale themes emerged from our findings that characterize the emotional experiences of academically achieving FGCS. Below, we present participants’ quotes illustrating the two themes by using randomly generated pseudonyms that align with participants’ self-reported gender identity.

### Theme 1: Feelings of Pride and Gratitude

The emotions of pride and gratitude were both salient in participant responses. Several FGCS in our study expressed feelings of pride, as expected in an achievement context. However, many participants also expressed feelings of gratitude, a prosocial emotion less expected in an individual achievement context. Some participants acknowledged not only the pride they felt, but also the support and advice they had received from other people and the sacrifices made by others that contributed to their achievements. This theme was derived from two subcodes in our data analysis: *Pride* and *Gratitude for Support from Others*. Please see Table 1 for an explanation of how each code was specifically defined and Table 2 for the frequency listing of the occasions when *Pride* and *Gratitude for Support from Others* occurred together as well as the number of times each was mentioned independently.

**Table 1:** Codes and definitions

Code	Definition
**Emotions**	
Pride	Mention of one’s pride or pride-related feelings (e.g., feeling a sense of accomplishment or achievement; feeling a boost in their conceptualization of self-worth)
Gratitude for Support from Others	Mention of one’s gratitude or gratitude-related feelings (e.g., grateful, appreciative, thankful) and/or acknowledgement of the support, opportunities, and/or advice they have received from other people in their lives
**Capitalization Regulation**	
Avoiding Showing Off and Negative Interpersonal Dynamics	Mention of one’s social sharing being influenced by not wanting to explicitly display (e.g., show off, brag) their academics and/or achievements due to potential interpersonal consequences
Extent of Similarity in Experiences	Mention of one’s social sharing being influenced by having similar or dissimilar experiences with others, including whether others have a similar identity, come from a similar socioeconomic background, or undergo similar academic experiences
Other People’s Socioeconomic Experiences	Mention of one’s social sharing being influenced by other people’s socioeconomic backgrounds or experiences, whether they be income-related, education-related (e.g., whether others received college degrees or are in academia), and/or occupation-related (e.g., being working-class or working in a profession or a specific job)
Difficulties Sharing within Family	Mention of any challenges, difficulties, or struggles in social sharing or in acknowledging their academic achievements with other family members
Difficulties Sharing within Academia	Mention of any challenges, difficulties, or struggles in social sharing or in acknowledging their academic achievements with other people in academia
Difficulties Sharing with Outsiders	Mention of any challenges, difficulties, or struggles in social sharing or in acknowledging their academic achievements with other people outside of family or academia

**Table 2:** Frequencies of codes by participants

Code	# of participants with code	Total # of participants	% of participants with code across total # of participants
**Emotions**			
Pride	18	32	56.25%
Gratitude for Support from Others	26	32	81.25%
Pride + Gratitude for Support from Others	14	32	43.75%
**Capitalization Regulation**			
Avoiding Showing Off and Negative Interpersonal Dynamics	28	32	87.50%
Extent of Similarity in Experiences	31	32	96.88%
Other People’s Socioeconomic Experiences	32	32	100%
Difficulties Sharing within Academia	32	32	100%
Difficulties Sharing within Family	32	32	100%
Difficulties Sharing with Outsiders	29	32	90.63%

This table displays the portion of participants who were identified with the respective codes at least once

#### Gratitude Co-occurring with Pride

Pride and gratitude are two positive emotions studied by scholars as having distinct and diverging social functions (e.g., Campos et al., 2013; Ritzenhöfer et al., 2019; Septianto et al., 2021) so it was surprising that they were at times occurring together within the same participant’s experience. One participant, Frances, described her prideful and grateful feelings within family as well as within academic contexts. When asked what kinds of emotions she feels when sharing her academic successes with academic peers, she said, “I feel proud, I feel grateful. If it's someone who’s a mentor, I feel very grateful for their support and their help… I think those two are big ones, I feel proud and very grateful.” She also acknowledged the support from family when asked what emotions she feels when sharing her successes with family members:

I feel it depends on the family members. With my mom, I feel proud because I want her to be proud of me. Just because she’s my mom and, you know, she made a lot of sacrifices for me to be able to have the life that I have. So that’s how I feel about that, and obviously it makes me emotional.

Another participant, Veronica, described that a significant moment of academic success for her was defending her dissertation proposal. When asked what made her decide to share with her academic peers, she responded: “Because I was proud and, you know, I was happy to share with my friends and I feel like they’re very supportive so I feel like it’s good to share your accomplishments… having people to celebrate with you is always motivating and nice.” Similar to Frances, Veronica’s quotes describe her pride and grateful feelings toward her academic friends for their support, describing that it is what makes her want to share.

When asked about a specific time she achieved academic success, Beatriz described the experience of defending her dissertation proposal, stating “I’m really proud of that moment,” and how it became a model of how to do it in her department. When asked what kinds of emotions she was going through after she accomplished this, Beatriz’s statements exemplified that both pride and gratitude for support from others were being experienced together:

I was emotional because I think of the sacrifices that have been made by my grandparents who come from a working-class family. My grandfather worked in factories in LA and then my grandmother picked produce, and they don’t know what the hell I'm doing. And I didn’t know what the hell I was doing either. So it’s these interesting, conflicting feelings, but it’s a lot of pride and it's a lot of emotion.

Beatriz also discussed the way she generally considers sharing her academic achievements with the people in her life. When specifically asked what emotions she generally tends to feel when considering sharing her academic successes with family members, Beatriz’s answer further demonstrated both pride and gratitude:

I am tremendously proud of what I’m doing. I will often think about my grandfather. He’s deceased, he's been gone for years. But I will often think about him as a way to keep me motivated, through his sacrifices. I’ll lean on what my family has done and what they’ve sacrificed to push me to be here, to have achievements.

The grateful appreciation that Beatriz and other participants feel seems to be rooted not only in having an understanding of the role of other people’s support in their achievement, but also in acknowledging the sacrifices made by family that created the possibility for those achievements.

#### Gratitude Occurring Independent of Pride

More than half of the study’s participants described how proud they were of their achievements, but what was even more surprising was the number of participants who demonstrated *Gratitude for Support from Others* regardless of pride (please see Table 2 for frequencies of *Pride* and *Gratitude for Support from Others*, respectively)*.* For example, at the end of our interview, Janette was asked if there were any people outside of her current academic environment and outside of family that she shares with about her accomplishments. She replied, “Professors from my undergrad who were my mentors…” because they “have been part of my academic journey for a very long time” and “they’ve been invested in me doing this since like undergrad.” When asked further if there are any factors that she usually considers when deciding to share her achievements with these mentors, Janette said:

I guess it’s just gratitude. I feel like when you share with someone who’s been a mentor, and who’s been invested in your success and seeing you grow, even if they don’t actually ask for it, I think it’s nice to update people who you think are in your corner and are invested in you to just let them know, Hey, it’s coming to fruition. Maybe it’s taken a lot of years [for me to reach this achievement] but it’s definitely [because of the] things that you’ve taught me or the feedback that you have provided in different stages… It has really helped me get here and so, ultimately, my academic achievement I don’t see as just a result of me and so I want to make sure that I share that academic achievement with people who have also helped me get there, whether it’s for that specific achievement or it’s more broadly for the journey.

Similarly, when asked how she usually feels when getting good news about her academics, Joanna replied:

If I applied to something, I feel like I would be most inclined to share that with other people in my cohort or mentors that helped me… and thanking them for helping me.

As another example, when asked about a specific time she achieved academic success, Patricia described the experience in her prior Master’s program of “finishing my thesis while also juggling applying to grad school” followed by receiving “the outstanding achievement for a Master’s student in the department.” When asked with whom she shared this achievement, she discussed the reasons why she shared with her sister that she had gotten the award and described what her sister's response to her sharing was:

It was really exciting to share that news because she sacrificed a lot for me. She kind of had to take care of me a lot when while she was doing her bachelor’s… Due to that, it was kind of exciting for me to show her, Hey, all those sacrifices that you made for me, you’ve taught me so much, and you’ve helped me in my education as well… [it was] kind of showing her the fruits of her sacrifice, I guess you could say.

She described it having made all her sacrifices worth it… and due to that, I just get really excited specifically to share things with her.

Patricia discusses the support she received from her sister within the context of her sister’s “sacrifices.” Patricia is demonstrating that the excitement over her individual achievement is embedded within her larger gratitude for what her sister sacrificed for her to reach the position she is in.

In addition to acknowledging the people who helped them reach their achievements, some participants also acknowledged the privilege and opportunities they have. When asked how he usually shares his academic successes with people outside of academia and outside of family members, Gustavo went into detail describing how he shares with his current romantic partner and specifically described wanting to show reciprocal support to his partner who has helped him:

I’m receiving all of this [help], but how am I showing up in return?... Am I supporting my partner’s work? Because she gives me so much, so I wonder, am I just getting all these wonderful questions [from her] and am I asking good questions for her, am I helpful?... I have a buffer just because I’m in a Ph.D. program that gives me access to all kinds of resources. You know, that’s a lot of privilege.

The participants in our study were proud over their achievements but additionally also made their gratitude shine through in the quotes they provided. Some of them expressed both their pride and gratitude within the same individual responses, but many others also expressed grateful feelings throughout their interviews that were not described within the same context as their pride. What this demonstrates is that both pride, expectedly, and gratitude, more unexpectedly, are relevant in an individual-focused context such as achievement.

### Theme 2: Capitalization Regulation

When describing how they capitalize on their academic achievements with other people, FGCS seemed to be engaging in what we are terming as *capitalization regulation*—which we define as the thoughtful consideration of how and with whom they share (i.e., capitalize on) good news across different social contexts (e.g., across their lower and higher social status worlds). Participants showed *capitalization regulation* through how they verbalized their challenges with sharing academic achievements across their family and university environments, often due to needing to describe them in ways suitable for the people they were capitalizing with or due to considering the potential for incurring negative social consequences. For example, participants expressed concerns that their sharing might come across as hubristic or that it might highlight the socioeconomic differences that exist (e.g., in family environments) when compared to the university.

The theme of capitalization regulation was derived from several subcodes in our data analysis that represented the various reasons participants gave for deciding whether to share achievements or not (i.e., *Avoiding Showing Off and Negative Interpersonal Dynamics*, *Extent of Similarity in Experiences*, and *Other People’s Socioeconomic Experiences* codes) as well as the subcodes that represented the challenges and difficulties that participants experienced in regard to sharing achievements across social contexts (i.e., *Difficulties Sharing within Family, Difficulties Sharing within Academia*, and *Difficulties Sharing with Outsiders* codes). Please see Table 1 for how each code was specifically defined and Table 2 for frequencies of the codes in the data.

#### Capitalization Regulation Within Family

Participants engaged in capitalization regulation within family contexts. As an example, Rodney considered how his sharing is influenced by whether other family members have had differing experiences to him (i.e., *Extent of Similarity in Experiences* code). Specifically, he discussed that he considers how to best share his achievements in ways that are suitable for family members from different socioeconomic backgrounds in order for them to have a clear understanding (i.e., *Other People’s Socioeconomic Experiences* code). When asked about a specific time he achieved academic success, Rodney stated, “I would say one of my favorite achievements that I had during undergrad was creating and teaching a class to other undergraduates.” When asked to describe with whom he shared this achievement and what the experience of sharing was like, Rodney responded with the below:

I did tell people within my family, and it’s funny because since a lot of folks in my family didn’t go to a four year institution… they were thinking like, Oh wow, my son, or my cousin is teaching a class at the university. But not really understanding the dynamics of it, since it was only a one unit pass/no pass class. It’s not like it was a four unit actual course. Sure, it did go on people’s transcripts. But in no way was I at the level of a graduate or faculty instructor, yet my family still kind of viewed me in that sense. So it was interesting to see their reaction to it as well… I kind of had to like backtrack and explain what it is exactly that I was doing and how it compares to what faculty research is on campus and kind of just breaking it down to them, showing them like this is just like the first step of what I would like to do in the future.

When asked to describe how he generally shares academic achievements with family members, even outside of the specific achievement he discussed, he stated:

I don’t really like flaunt different things that I’m doing, but if somebody asks me how school is going, or what I’m up to, I’ll go ahead and share with them.

In the quote above, Rodney expresses that he does not always share (i.e., *Difficulties Sharing within Family* code) because he does not want to “flaunt” (i.e., *Avoiding Showing Off and Negative Interpersonal Dynamics* code). When probed further and asked why he mentioned that he does not like to flaunt the information, he replied with:

Well first off, it comes with a lot of description because I know that whatever I’m describing, they might not necessarily fully understand it. And I don’t want to just give them partial information. So I guess it’s kind of taxing in that way. But also, some folks in my family didn’t have the opportunity to go to college so I don’t like to flaunt the fact that I am in university. I’m just not trying to highlight those differences.

Through these quotes, Rodney highlights his experience sharing with family members that involves explaining his achievements to them in ways that can be relatable to them. He understands that they might not always understand his achievements. He also describes not wanting to highlight the socioeconomic differences that exist across family members; he describes the academic opportunities he had that other family members did not.

Frances also seems to be regulating her capitalization for similar reasons. When asked what kind of factors she considers when deciding to share with family, she replied with:

Things like awards with money are very easy to share, because it’s very obvious the benefit, especially because my mom was a single mom, we were in a lower income household and community. And so things with money have a very obvious purpose, right? Like it pays you… whereas other things, my mom doesn’t know... Showing her quantitative papers I’ve written, she will not understand and I’m not in a place where… I don’t want to have to show her things and then make her feel dumb about it, because she’s not dumb, she’s not a dumb person. And so I tell her I wrote a paper and I’ll show her the paper but I don’t ask her about it after I just showed it to her. I want to share the information without her feeling like she’s going to be grilled about it in any way because I don’t want her to feel like that.

Frances acknowledges how the socioeconomic background and experiences of her mother may not lead her to fully understand her work. Specifically, she wants to make sure her mother is not self-critical if she does not understand the work and is therefore thoughtful about how she presents it. Joanna is similarly careful about how her sharing may be perceived by family who have not had the same opportunities she has. When asked what emotions she feels when she considers sharing her achievements with family members, she replied:

There’s definitely excitement going on there. But I guess, sometimes when sharing with family… there’s sometimes hesitancy too because I have family that’s around the same age as me that didn’t go into college at all, or schooling at all. So sometimes when you share stuff, it feels like you’re bragging, or they make it seem like you’re just bragging all the time. So sometimes there is some hesitancy there. But for the people that I always want to share with, like my parents, yeah it’s excitement.

Joanna does seem to feel excited about sharing with her parents, but it seems like she does find it challenging to sometimes share with other family members that are closer to her age who did not proceed in academia the way she did. When probed further and asked if she could go into more detail as to why she mentioned the bragging, Joanna described that her achievement of graduating from her undergraduate institution:

…felt like some type of bragging to some people, especially in the context of when I graduated… I felt like I couldn’t really share on social media things that I was doing because so many things were going on in the world… So I felt like, oh, if I’m sharing this and flexing on everybody, then that's kind of going to be seen as bragging in a way.

Through these quotes, Joanna highlights her thoughtfulness by considering how the context of real-world events might impact the way that her social sharing of personal good news might be perceived by family members. She even mentions that she could not really share on social media, hinting that her regulation may extend to individuals outside of just family members (i.e., *Difficulties Sharing with Outsiders* code).

Another participant, Terrance, described mixed emotions when asked how he feels when he considers sharing his achievements with family members:

With my parents, it’s usually for the most part excitement and a little bit like, Okay, now please don’t go tell everyone. With some of my other family… they always ask, Have you won any of this? Or have you done this or that? But it’s usually a little nerve-racking because I know they understand even less what’s going on. So it’s just like, How do I say these things, again, without feeling like I’m bragging?”

Similar to other participants, Terrance considers that his family may not fully understand the achievement he wants to share as they have had very different socioeconomic experiences from him. He considers how he can best share it without coming across as “bragging.”

#### Capitalization Regulation Within Academia

Participants also demonstrated capitalization regulation within their academic environment. Similar to the recurring concern participants had with family members, many participants described not wanting to “brag” with academic peers (i.e., *Avoiding Showing Off and Negative Interpersonal Dynamics* code). For example, Alejandra mentioned that she had shared a specific achievement, passing the qualifying exams in her department, with her friends in the department; when asked what the experience of sharing this achievement was like with all these people, she described that it was:

…difficult because I wanted to express that I felt very happy that I was able to pass these qualifying exams… but at the same time, I didn’t want to sound like I was bragging.

Another participant, Nora, also expressed a concern with bragging. When asked what a sharing moment generally looks like for her, one in which she is talking to someone and is trying to share that she has achieved or has had success in some academic endeavor, Nora responded with the statement below:

It’s [my sharing] still a subtle thing, because I know in academia to advocate for yourself [is the norm], but part of me is like I don’t want to brag, but I don’t know how to humble brag, and I don’t want to stay mute, either.

Nora understands that the social norm in academia is to share achievements, but she finds it challenging to advocate for herself without “bragging” (i.e., *Difficulties Sharing within Academia* code). She further describes how her socioeconomic background influences the way she navigates how she shares within her academic environment:

I think as a first gen student it’s like trying to learn the ropes… it’s like one of those faux pas that I didn’t really come to understand at the point until a few months ago, but now I’m just like, Okay so what else should I know? How else should I navigate academia? So I don’t come off as someone that’s like, Hey this is my CV, every single minute of the day or whenever I have an academic conversation with someone.

Terrance also finds it very challenging to share within academia. In addition to the considerations he has sharing with family members (as outlined in the previous section), when asked what emotions he feels when he considers sharing his achievements with academic peers:

To be honest, I always get extremely nervous… I’ll get nervous anxiety, and question, Do I really want to tell people? Should I tell people this? Is it something to celebrate?... There are these other pressures that weigh on me, whether back to the showing off, or do people care, do I want the attention, stuff like that.

Another participant, Marcos, also seems to be regulating his capitalization. After describing his academic achievement as being a recent article he had been able to publish without any major revisions requested from reviewers, he stated that he had not shared it with his group of academic friends, whom he described as graduate students who were in his department but a few years below him. Specifically, he said:

They knew that I submitted it and I told them that I got accepted, but I never told them the fact that I never got edits… They were surprised that it was published quite quickly, that was recognized fairly immediately. But I didn’t share that element, that it was accepted without edits, and I sort of feel like I have to keep that secret in some sense.

When asked to further elaborate on why he wanted to keep that as a secret, he continued:

I think the main reason is, it’s sort of like navigating with cohort members or underclassmen… I don’t want them to feel like imposters. And so I don’t give them this thing that I know is very unattainable for a lot of people, this skill of writing so well or something like that. And in my opinion, I would be doing a disservice in some sense if I were to gloat or whatever. Even though technically it might not be gloating, it’s just you know, sharing a little achievement, but I think you could take it too far and it might result in more negatives than positives…

Through his quotes, Marcos suggests that he understands the social consequences that may ensue by sharing his full achievement. He describes that he did not share the full details of his accepted publication with other graduate student peers so that they would not feel like “imposters.” He was thoughtful in what he chose to share (his article being accepted) and in what he chose to not share (that it did not come with edits). Similar to the other participants, Marcos’ quotes highlight his capitalization regulation within the academic context.

#### Capitalization Regulation Beyond Family and Academia

While most participants described their capitalization processes within family and academic environments, some also described what their sharing was like beyond just those two social contexts. For example, when asked if she shares across different types of platforms with academic peers, Beatriz described that she does not share through social media because of how it might affect not only her family but also her friends *outside* of academia (i.e., *Difficulties Sharing with Outsiders* code*)*. She stated:

I feel conflicted about social media period. I have many colleagues who do document their entire process on social media, and I know there’s value to that… especially if you’re from an underrepresented or historically marginalized group. I think why I just [hesitate] is because I have a lot of people on my social media, who are my friends, that are family, like grandparents, aunts, uncles, cousins. And I have a lot of friends who either serve working class communities or are working class or are from historically marginalized groups themselves and are in a working class occupation. And I just hesitate on what it looks like for me to be showing these things that I’m doing. I question myself like, why are you doing that, you know? Because there’s tremendous privilege that comes with what I am doing. And that privilege is really conflicting, it causes a lot of conflicts internally in me, because I don’t know a lot of people who know exactly what I’m doing and understand. It kind of makes me feel bad. So I’ve really refrained from sharing stuff on social media outside of everyday normal stuff.

Beatriz’s quote exemplifies the various reasons participants gave throughout the study for deciding whether to share achievements or not (i.e., *Avoiding Showing Off and Negative Interpersonal Dynamics*, *Extent of Similarity in Experiences*, and *Other People’s Socioeconomic Experiences* codes). Beatriz has awareness of the privilege that she has compared to others who do not have the same opportunities, as exemplified in the quote above and throughout her quotes in the previous sections highlighting her gratitude. She mentions that there is “value to that” process of sharing if being from an underrepresented group, but even still she feels reluctance to share because of what it would look like to others who are not in her same position. Her quote explaining why she chooses not to share through social media is an example of the capitalization regulation that she is actively partaking in.

## Discussion

This study explored how individuals experiencing upward social mobility via higher education navigate the emotion-laden experience of academically achieving across their family and university environments. Our findings show that academic achievement elicits complex positive emotions and capitalization regulation, the thoughtful consideration of how and with whom one shares good news across social contexts.

### Pride and Gratitude

While recent capitalization work has highlighted the interdependent emotion of gratitude (e.g., Gray et al., 2024), our findings are novel in showing that gratitude is salient in an academic achievement context. It can co-occur and even occur regardless of pride, the self-focused and expected emotion in this context. Both pride and gratitude were prevalent for participants through how they described their achievements as being tied to their collective—the way others supported them that led to their achievements. Many participants acknowledged the sacrifices made by family members that made their achievements possible. They demonstrated an understanding that older family members had to give something of themselves to help pave the way for future generations like them to achieve and rise in the social mobility ladder. Our findings thus show that a prideful experience can also be experienced as a grateful one, further establishing the contribution of the interdependence theory of emotion (see Uchida et al., 2022) and showcasing how discrete emotions usually categorized as “independent” or “interdependent” can be re-contextualized based on the social contexts that individuals navigate.

### Capitalization Regulation

We introduce the term *capitalization regulation* to capture the thoughtfulness shown by FGCS in how they capitalize across their family and university environments by considering the negative consequences that their good news may elicit. Within family environments, FGCS want to share in ways that family members will understand and that will not highlight existing SES differences, a concern that aligns with family achievement guilt (Covarrubias & Fryberg, 2015) and how FGCS’ transition into university makes salient the socioeconomic discrepancies that exist (Covarrubias & Fryberg, 2015; Stephens et al., 2012). Within both family and university environments, FGCS are concerned that their sharing may be perceived as “bragging,” a consequence often described in the context of pride (Kalokerinos et al., 2014; Lange & Crusius, 2015) that aligns with why college students may not share positive experiences (Derlega et al., 2011).

The capitalization regulation shown by FGCS may be explained by literature suggesting that individuals of lower, compared to higher, SES show more consideration of others (Anderson et al., 2015; Galinsky et al., 2006; Keltner et al., 2003; Kraus et al., 2010; Piff & Moskowitz, 2018; Piff et al., 2010, 2012), therefore fitting within the interdependence theory of emotion that promotes emotions as collective experiences (Uchida et al., 2022). It may be a socio-emotional strength that allows individuals to foster interpersonal relationships across distinct status environments by regulating themselves in social circumstances when it may not be appropriate to share.

### Limitations and Strengths

A limitation of our study is potential selection bias. While FGCS at the graduate level may have deeper insight into the SES-shifting experience, they may have acculturated to the university’s independent norms and thus their experiences may not be representative of all FGCS. Still, graduate students are important to study because their well-being is a salient concern (e.g., Charles et al., 2022). Additionally, while some participants described how their sharing was impacted by the COVID-19 pandemic (interviews occurred between December 2020 and September 2021), we did not ask standardized pandemic-focused questions to all participants. However, capitalization regulation is still relevant as people’s social lives have transitioned to the new normal.

Our study does have notable strengths. First, utilizing qualitative methodology allowed for rich insight into the complexities brought forth by academic achievement that would not have been possible by relying solely on existing quantitative paradigms. Qualitative methodology was necessary to uncover the construct of capitalization regulation; only through in-depth interviews were we able to capture the thoughtful consideration that participants have regarding their social sharing. Additionally, our study’s merging of different psychological literatures—capitalization, SES, and FGCS—has implications for future work on upward social mobility and interpersonal emotion regulation.

### Future Directions

While the current study observed pride and gratitude together when the thematic content of responses was examined holistically, further quantitative studies will be necessary to determine if true covariation occurs between pride and gratitude in the context of academic achievement. Examining this potential covariation can lead to an understanding of the circumstances wherein expressing both emotions together may accrue academic benefits (i.e., better learning and achievement outcomes; King & Datu, 2018; Shi et al., 2022; Villavicencio & Bernardo, 2013; Wolgast et al., 2022) without pride’s adverse social consequences. Studies should also examine whether the potential covariation between pride and gratitude varies by SES or ethnic background. We did not have large subsamples of non-Latino/a/x/e groups to explore this in our own study, but examining Asian and Latino/a/x/e differences is important because most work that has established emotions as interdependent has usually focused on Asian cultures (e.g., Uchida et al., 2022). Not only do Latino/a/x/e cultures have their own form of interdependent ideals (e.g., convivial collectivism) that differ from those of Asian cultures (Campos & Kim, 2017), but newer studies are starting to suggest that Latino/a/x/e cultures emphasize both interdependent *and* independent values (Krys et al., 2019, 2022; Vignoles et al., 2016). Studies have begun to focus on Latino/a/x/e students’ interdependent emotional processes (e.g., gratitude; Wang et al., 2023) and continuing to examine them will yield further insight into their affective experiences.

Similarly, studies should examine what aspects of capitalization regulation vary by SES or ethnic background, especially as some concerns expressed by our participants (e.g., hindering interpersonal relationships) are echoed among Asian interdependent cultures (Choi et al., 2019). While some concerns may be shared regardless of backgrounds (e.g., “bragging”), concerns over highlighting SES differences may be particularly salient among groups for whom SES inequalities loom large. Future work is crucial because while capitalization regulation can be viewed as a potential socio-emotional strength that carries interpersonal benefits (e.g., avoiding envy), regulating sharing might hinder the positive affect associated with capitalizing (Gable et al., 2004; Langston, 1994) and lead FGCS to forego beneficial elevations in long-term social status. To assess capitalization regulation further, researchers should draw from previous studies (Gable et al., 2004; Lambert et al., 2013) and leverage approaches that include assessing sharing behaviors through multiple methods (e.g., laboratory experiments or ecological momentary assessment).

## Conclusion

Exploring the academic achievement experience of first-generation college students through qualitative methodology yielded new insight into emotion processes, including feelings of pride and gratitude and engagement in capitalization regulation, a potential socio-emotional strength that leads individuals to consider the social consequences that may ensue by sharing achievements across differing SES worlds. Our work makes contributions to the affective science literature on interpersonal emotion regulation and social class by generating new knowledge on the socio-emotional strengths that underrepresented students may acquire when building bridges across social worlds. Our findings open new research avenues that can encourage scholars to continue examining how social status and ethnic culture influence the way individuals move across the social hierarchy, thus hopefully informing efforts to better assist SES-shifting groups (e.g., in the university) to thrive as they move through their two social worlds. Altogether, our work informs societal understanding of the complex experiences associated with undergoing upward social mobility through educational advancement while opening doors that can benefit diverse and underrepresented groups.

## Supplementary Information

Below is the link to the electronic supplementary material.

Supplementary file1 (DOCX 33 KB)

Supplementary file2 (DOCX 41 KB)

## Footnotes

1 See Supplemental Material for racial/ethnic backgrounds as self-reported by each participant.

2 See Supplemental Material for interview guide.

## Additional Information

### Data Availability

 Due to the qualitative nature of the paper, data (i.e., transcribed interviews) will not be made available in a repository. Research material (i.e., interview guide) and participant ethnic/racial backgrounds are available as supplemental material.
