# Supplementary material for: Show Your Pride? First-Generation College Student Experiences with Academic Achievement
Source: Affect Sci. 2025 Jun 6;6(3):414–27. doi: 10.1007/s42761-025-00309-w (PMC12579612; doi:10.1007/s42761-025-00309-w)
Supplement: Supplementary file 1 — Supplementary file1 (DOCX 33 KB) [file 42761_2025_309_MOESM1_ESM.docx]

**Show Your Pride?**

**First-Generation College Student Experiences**

**with Academic Achievement**

Hugo Sanchez Hernandez^1^, Jorge Castro Jr^2^, and Belinda Campos^3^

^1^Department of Psychology, University of California, Los Angeles, California, United States

^2^Department of Psychological Science, University of California, Irvine, California, United States

^3^Department of Chicano/Latino Studies, University of California, Irvine, California, United States

**Author Note**

Hugo Sanchez Hernandez
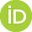
 <https://orcid.org/0000-0002-1818-6953>

Jorge Castro Jr
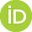
 <https://orcid.org/0009-0002-7992-6983>

Belinda Campos
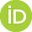
 <https://orcid.org/0000-0001-5650-971X>

Author note: Correspondence concerning this article should be addressed to Hugo Sanchez Hernandez, Department of Psychology, University of California, Los Angeles, 1285 Franz Hall, Box 951563, Los Angeles, California, United States, 90095. Email: [hugosh@psych.ucla.edu](mailto:hugosh@psych.ucla.edu).

This research was conducted while the first author was in the Department of Psychological Science, University of California, Irvine, 4201 Social and Behavioral Sciences Gateway, Irvine, California, United States, 92697-7085.

Journal submission: **Affective Science**

| **Participant ID** | **Gender** | **Pseudonym** (for presentation of findings) | **Racial/Ethnic background** |
| --- | --- | --- | --- |
| **95101** | F | Victoria | Latinx |
| **95102** | F | Dolores | Xicana/ Mexican-American |
| **95103** | F | Julia | Chicanx/ Mexican American |
| **95104** | M | Rodney | Arab American |
| **95105** | F | Patricia | Latino/a |
| **95106** | F | Sophia | Asian/Vietnamese |
| **95107** | F | Nora | Southeast Asian, Lao-American |
| **95108** | M | Arturo | Hispanic |
| **95109** | Non-binary/transmasculine | Gustavo | Latinx |
| **95110** | F | Joanna | Hispanic/Mexican |
| **95111** | F | Lilliana | Mexican-American |
| **95112** | F | Beatriz | Native American and Chicanx |
| **95113** | M | Hector | Latino/Mexican |
| **95114** | F | Ruby | White/ Polynesian |
| **95115** | Preferred not to say | Lauren | Did not indicate |
| **95116** | F | Janette | Latina |
| **95117** | F | Bertha | Latina |
| **95118** | F | Frances | Biracial |
| **95119** | F | Veronica | Latina |
| **95120** | F | Janelle | White/Mexican |
| **95121** | M | Francisco | Mestizo (Colombian) |
| **95122** | F | Viola | Peruvian |
| **95123** | F | Olga | Latina/Mexican American |
| **95124** | F | Nadine | Non-Hispanic White |
| **95125** | M | Bronson | Asian/Chinese |
| **95126** | F | Melody | Turkish |
| **95127** | M | Wilbur | White |
| **95128** | M | Ivan | Latino |
| **95129** | M | Terrance | Native American |
| **95130** | M | Marcos | Hispanic/Peruvian |
| **95131** | M | Julio | Latino |
| **95132** | F | Alejandra | Latinx |
