# Supplementary material for: Show Your Pride? First-Generation College Student Experiences with Academic Achievement
Source: Affect Sci. 2025 Jun 6;6(3):414–27. doi: 10.1007/s42761-025-00309-w (PMC12579612; doi:10.1007/s42761-025-00309-w)
Supplement: Supplementary file 2 — Supplementary file2 (DOCX 41 KB) [file 42761_2025_309_MOESM2_ESM.docx]

**Show Your Pride?**

**First-Generation College Student Experiences**

**with Academic Achievement**

Hugo Sanchez Hernandez^1^, Jorge Castro Jr^2^, and Belinda Campos^3^

^1^Department of Psychology, University of California, Los Angeles, California, United States

^2^Department of Psychological Science, University of California, Irvine, California, United States

^3^Department of Chicano/Latino Studies, University of California, Irvine, California, United States

**Author Note**

Hugo Sanchez Hernandez
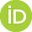
 <https://orcid.org/0000-0002-1818-6953>

Jorge Castro Jr
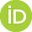
 <https://orcid.org/0009-0002-7992-6983>

Belinda Campos
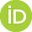
 <https://orcid.org/0000-0001-5650-971X>

Author note: Correspondence concerning this article should be addressed to Hugo Sanchez Hernandez, Department of Psychology, University of California, Los Angeles, 1285 Franz Hall, Box 951563, Los Angeles, California, United States, 90095. Email: [hugosh@psych.ucla.edu](mailto:hugosh@psych.ucla.edu).

This research was conducted while the first author was in the Department of Psychological Science, University of California, Irvine, 4201 Social and Behavioral Sciences Gateway, Irvine, California, United States, 92697-7085.

Journal submission: **Affective Science**

**INTERVIEW GUIDE**

Hi, my name is [LEAD AUTHOR NAME]. Thank you very much for being here. As we have already discussed, I am conducting a study on the academic experiences of graduate students. This interview might take anywhere between 30 – 60 mins, but before we begin, I want to ask you a few things to confirm your eligibility again.

Eligibility:

1) Are you a current graduate student?

2) Has a parent/guardian of yours obtained a 4-year college degree?

Study Information Sheet (Online):

We can now go over some additional information about this interview. The first is the study information sheet that I sent to you ahead of time. Once we have gone through this information, you can indicate whether you offer your consent to participant in this study. I have sent you an email with a link to view this sheet online. Please let me know that you have received it and open the link so we can start going through it together.

*Go through Study Info Sheet*

Once you have completed the consent section let me know and we can continue.

Video Release Form (Online):

We can now go over the video release form. *If asked:* *Option 1 in the video release form is what you have already agreed to in the study information sheet and is the minimal consent needed to participate in the study.*

*Go through Video Release Form*

------------------------------------------------------------------------------------------------------------------------------------

Before I start the recording, let’s go ahead and edit your name on Zoom so there is no chance of it appearing in the recording. You can click on Participants and edit your name directly on there. Please replace your name with the participant number that I am going to give you: **XXX.** **Please try to not use any actual names during the interview of the people you are referring to, including your own.** **Also please try not to use any specific names of the departments or majors you are in within [UNIVERSITY NAME]. For example, if you want to mention something that happens within your department please keep the specific department name vague**. Please note that once I start the recording, Zoom will prompt you for your consent. There have been some technical difficulties that have occurred in the past, such as the Zoom on the remote computer crashing. If that occurs please give me a minute or so to start it back up again and we can resume where we left off.

***START RECORDING*****

Okay, let me check that everything is recording correctly.

Now that we have gone through the consent and video release forms, can you again verbalize your consent to participate in this study so that it is captured on the recording?

Warm-Up:

The purpose of this study is to find out more about the academic experiences of graduate students. I want to talk with you about how you share your academic achievements or success with the people around you. There are no right or wrong answers, you are the expert in this. Anything you feel comfortable sharing would be greatly appreciated. I anticipate that the interview might be anywhere between 45-60 min, does that still work for you? If we find ourselves in the middle of an important point, we might want to go over the allotted time, but that will be up to you because you’ve only agreed to a 60 minute interview at most. During the interview, please let me know if you need me to define or explain anything about the questions I ask.

**This following includes a list of all potential questions to ask. Time might not permit all questions to be asked, and questions will be asked as necessary. If additional information comes up during interview that warrants further probing and questioning that is not included here, those will be asked.**

**Interview Questions**

**How do you usually feel when you are given good news about something related to your academics?**

**For you, what is an academic achievement or academic success?**

**What kind of academic achievements are important to you?**

- - **Probe***: Can you describe why?**

**Are there any kind of academic achievements that are NOT important to you?**

- - **Probe***: Can you describe why?**

**RQ 1: Can you describe to me a time you performed well academically or achieved academic success?**

- - ***[If asked: By academic success, I mean any aspect of your educational experience where you performed well that made you feel positively. For example: It could be anything from a good grade you received, a project you did really well in, research or academic presentation that you did really well in, a scholarship or award you received, a high GPA that made you feel happy, etc.]***
  - **Probe***: What kind of academic success do you have in mind?**
  - **Probe***: Can you tell me more about it - what was that experience like?**
    - **How did you feel when you knew you ___ (***fill in with the success****)?***

*Prompt**: Can you describe to me - with whom did you share this achievement?**

- - ***If friend, ask: Is this a friend/partner you know academically (at school) or friend/partner you know outside of academics?***
  - **Probes***: Can you tell me more about this sharing - what was that experience like?**
    - **How did you share?**
      - **In person? Through social media? Online conversation? Phone call?**
    - **What made you decide to share?**
    - **How did the person/people respond?**
  - **Probes***: Is this typically with whom you share your academic successes with?**
    - - **Why would you say you share with them most of the time?**
      - **If NO**
        - **With whom do you typically share your academic successes with?** [go to appropriate context section after this]
  - ***If NO sharing***
    - **Why did you not share this with anyone?**
      - **What made you decide not to share?**

[*If they start talking about a peer/partner on campus do rest of contexts in order; if they mention a family member or non-academic peer/partner go to those sections first*]

**Academic Peer contexts of sharing:**

**RQ 2a: How do you usually share academic successes with academic peers (*[If asked: e.g., classmates, peers, teachers, advisors, romantic partners- anyone that you know in an academic context*)?** *[Return to this section depending on what context they talked about first]*

- - *Prompt**: What do you think about, or what factors do you consider, when deciding to share or when you consider sharing with academic peers?**
  - *Prompt**: What kind of emotions do you feel when you share or consider sharing academic successes with academic peers?**
  - *Prompt**: Do you share your academic successes differently depending on who the academic peer is?**

**Probes*

- - - **If so, how do you share things differently?**
    - **If so, how do you usually decide which academic peers you are going to share your academic successes with?**
    - **Which people do you share with vs. which people do you not share with?** *(asking for their roles, like classmates or teachers, rather than specific names)*
  - *Prompt**: Does your sharing with academic peers depend on other things, maybe external factors? (e.g., The quarter you are in, classes you are taking, the time of the day, where you are?)**
  - *Prompt**: Are there certain academic peers you share with and others you do not?**
  - *Prompt**: Do you share across different types of platforms with academic peers? (e.g., in person, through social media, phone call)**
    - **How so?**

**Family contexts of sharing:**

**RQ 2b: How do you usually share academic successes with family members (*If asked: e.g., anyone who you are biologically related to or who you consider to be part of your family, can be immediate family like siblings or can be distant family like a great uncle or aunt, or even a partner if you are married to them – anyone that you know in a family context)?*** *[Return to this section depending on what context they talked about first]*

- - *Prompt**: What do you think about, or what factors do you consider, when deciding to share or when you consider sharing with family members?**
  - *Prompt**: What kind of emotions do you feel when you share or consider sharing academic successes with family members?**
  - *Prompt**: Do you share your academic successes differently depending on who the family member is?**

**Probes*

- - - **If so, how do you share things differently?**
    - **If so, how do you usually decide which family members you are going to share your academic successes with?**
    - **Which people do you share with vs. which people do you not share with?** *(asking for their roles, like mother, sister, cousin, aunt rather than specific names)*
  - *Prompt**: Does your sharing with family members depend on other things, maybe external factors? (e.g., The quarter you are in, classes you are taking, the time of the day, where you are?)**
  - *Prompt**: Are there certain family members you share with and others you do not?**
  - *Prompt**: Do you share across different types of platforms with family members? (e.g., in person, through social media, phone call)**
    - **How so?**

**Non-specific contexts of sharing:**

**RQ 2c: How do you usually share academic successes with people outside of academia who are not your family? - For example, hometown friends who are not in your college, romantic partners you are not married to who you know outside of an academic context, neighbors who you know from outside of your school - anyone that you know outside of an academic or family context** *[Return to this section depending on what context they talked about first]*

- - *Prompt**: What do you think about, or what factors do you consider, when deciding to share or when you consider sharing with people outside of academia?**
  - *Prompt**: What kind of emotions do you feel when you share or consider sharing academic successes with people outside of academia?**
  - *Prompt**: Do you share your academic successes differently depending on who the person outside academia is?**

**Probes*

- - - **If so, how do you share things differently?**
    - **If so, how do you usually decide which people outside of academia you are going to share your academic successes with?**
    - **Which people do you share with vs. which people do you not share with?** *(asking for their roles, like neighbor, hometown friend, rather than specific names)*
  - *Prompt**: Does your sharing with people outside of academia depend on other things, maybe external factors? (e.g., The quarter you are in, classes you are taking, the time of the day, where you are?)**
  - *Prompt**: Are there certain people outside of academia you share with and others you do not?**
  - *Prompt**: Do you share across different types of platforms with people outside of academia? (e.g., in person, through social media, phone call)**
    - **How so?**

*Prompt**: Are your sharing experiences (i.e., how you share, what you share) different now than they were in the past? *Perhaps different now than they were when you were an undergraduate student?***

- - **In what way are they different now than they were when you were an undergrad student?**

*Prompt**: Are there any other people that you share with that we have not discussed (e.g., if applicable: any other family members, romantic partners, etc)?**

- - - ***If they mentioned having romantic partner and it has not already been discussed:* How would you say you share with your partner? Do you share most things, share everything?**

**Debriefing**

- **Is there anything else about the experience of sharing your academic achievements or success that you would like to mention that we have not discussed?**

Great. I will now stop the recording before finishing the session.

***STOP RECORDING*****

Thank you very much for your time. As a reminder, the audio- and video-recording of this conversation will be transcribed, and any identifying names will be replaced with pseudonyms so that no participant names are linked to the data.

You will receive an email from me within the next few days with your Target gift card. Do you have any final questions?
